# The Evolution of Advanced Molecular Diagnostics for the Detection and Characterization of *Mycoplasma pneumoniae*

**DOI:** 10.3389/fmicb.2016.00232

**Published:** 2016-03-08

**Authors:** Maureen H. Diaz, Jonas M. Winchell

**Affiliations:** Pneumonia Response and Surveillance Laboratory, Respiratory Diseases Branch, Division of Bacterial Diseases, National Center for Immunization and Respiratory Diseases, Centers for Disease Control and Prevention, AtlantaGA, USA

**Keywords:** *Mycoplasma pneumoniae*, molecular diagnostics, molecular epidemiology, molecular characteristics, whole genome sequencing

## Abstract

Over the past decade there have been significant advancements in the methods used for detecting and characterizing *Mycoplasma pneumoniae*, a common cause of respiratory illness and community-acquired pneumonia worldwide. The repertoire of available molecular diagnostics has greatly expanded from nucleic acid amplification techniques (NAATs) that encompass a variety of chemistries used for detection, to more sophisticated characterizing methods such as multi-locus variable-number tandem-repeat analysis (MLVA), Multi-locus sequence typing (MLST), matrix-assisted laser desorption ionization–time-of-flight mass spectrometry (MALDI-TOF MS), single nucleotide polymorphism typing, and numerous macrolide susceptibility profiling methods, among others. These many molecular-based approaches have been developed and employed to continually increase the level of discrimination and characterization in order to better understand the epidemiology and biology of *M. pneumoniae*. This review will summarize recent molecular techniques and procedures and lend perspective to how each has enhanced the current understanding of this organism and will emphasize how Next Generation Sequencing may serve as a resource for researchers to gain a more comprehensive understanding of the genomic complexities of this insidious pathogen.

## Introduction

*Mycoplasma pneumoniae* is a common cause of respiratory infections in all age groups worldwide (Waites and Talkington, 2004; Atkinson et al., 2008; Waites and Atkinson, 2009; Winchell, 2013). *M. pneumoniae* infections vary dramatically in clinical presentation, ranging from mild, self-limiting upper respiratory symptoms to radiographically confirmed pneumonia requiring hospitalization (Waites and Talkington, 2004; Atkinson et al., 2008). In some cases, infection may result in severe clinical syndromes involving other organ systems (Waites and Talkington, 2004; Atkinson et al., 2008; Narita, 2010; Olson et al., 2015; Magun et al., 2016). Localized outbreaks of *M. pneumoniae* have been reported frequently, especially in closed settings, and transmission between household contacts is known to occur (Foy et al., 1966; File et al., 1998; Walter et al., 2008; Waites and Atkinson, 2009; Winchell, 2013). The long incubation period of up to 3 weeks and prolonged shedding after infection allows outbreaks to often go unnoticed and extend for long periods of time (Foy et al., 1966; File et al., 1998; Hammerschlag, 2001; Atkinson et al., 2008; Nilsson et al., 2008). Larger community-wide outbreaks are frequently not identified or are recognized late. This underrecognition is due, in part, to a lack of pathogen-specific testing for mild respiratory illness in the primary care setting. However, increased incidence of *M. pneumoniae* infections in Europe, Asia, and the United States has been reported since 2010 (Lenglet et al., 2012; Diaz et al., 2015b; Kim et al., 2015). Such epidemic seasons of *M. pneumoniae* have been reported to occur every 4–7 years (Foy et al., 1979; Waites and Talkington, 2004; Atkinson et al., 2008; Winchell, 2013).

Despite the diversity and complexity of the clinical and epidemiological characteristics of *M. pneumoniae* infections, the bacterium itself is one of the smallest and simplest known organisms capable of living outside of a host cell. The genome of *M. pneumoniae* is approximately 800 kb in size, maintaining a set of approximately 700 annotated protein-coding genes (Guell et al., 2009; Lluch-Senar et al., 2015; Xiao et al., 2015) plus additional non-coding RNA genes (Dandekar et al., 2000; Xiao et al., 2015). The reduced genome is deceivingly complex as it contains tracts of tandemly repeated sequences at numerous loci (Degrange et al., 2009) and has been proposed to utilize sophisticated transcriptional regulatory mechanisms and antigenic variation to control gene expression (Guell et al., 2009; Spuesens et al., 2009, 2011; Citti et al., 2010). Variation of surface-exposed immunogenic epitopes, including the P1 adhesion molecule, have been reported (Spuesens et al., 2009, 2011) and may be a driving force for the cyclic occurrence of epidemic seasons of *M. pneumoniae* infections (Dumke et al., 2004).

Methods for identification of *M. pneumoniae* infection include culture, serological analysis, or molecular detection of pathogen-specific antigen(s) or nucleic acid. Culture is a definitive method for diagnosis of *M. pneumoniae*, and ongoing maintenance of a collection of clinical isolates is critical for monitoring trends in the epidemiology of this organism. However, culture is slow and requires specialized media and trained personnel, and, most importantly, recovery of isolates is highly variable and may be low, even in specialized laboratories (Ieven et al., 1996; Dorigo-Zetsma et al., 1999; Morozumi et al., 2004; Thurman et al., 2008). Serological analysis has proven problematic for the specific identification of *M. pneumoniae* infection due to poor diagnostic sensitivity and specificity and the requirement for paired acute and convalescent sera, and it does not allow for characterization of the *M. pneumoniae* strain causing the infection (Beersma et al., 2005; Nir-Paz et al., 2006; Thurman et al., 2008). Neither culture nor serology is practical for rapid detection of acute infection, limiting the utility of these methods to retrospective investigations.

Molecular methods for nucleic acid or antigen detection have emerged as the primary techniques for identification of *M. pneumoniae* in surveillance programs. However, adoption of these methods has lagged in the clinical setting in the United States with many physicians continuing to rely on serological tests or opting for no pathogen-specific testing in concordance with the guidelines from the Infectious Diseases Society of America (IDSA) for the treatment of community-acquired pneumonia (CAP; Mandell et al., 2007; Bradley et al., 2011). Beyond the methods for detection of *M. pneumoniae* in clinical specimens, numerous molecular methods have been developed in recent years that exploit the limited genomic diversity of *M. pneumoniae* isolates in order to characterize isolates for epidemiological purposes, although no clear correlation of strain type with clinical presentation, disease severity, or patient outcome has been identified to date. The emergence of macrolide resistance in this species has also spurred the development of molecular methods for determining susceptibility to this frontline antibiotic to improve appropriate prescribing. More recently, whole genome sequencing (WGS) has emerged as a more accessible and thorough approach for investigating the biological and epidemiological characteristics of *M. pneumoniae*. This review summarizes the various molecular methods for both detection and characterization of *M. pneumoniae* with a focus on WGS and the potential of this approach to transform the field in coming years.

## Detection of *M. pneumoniae* Using Nucleic Acid Amplification Techniques (NAATs)

Respiratory infections, including CAP, may be caused by a wide variety of pathogenic microorganisms that are indistinguishable by clinical evaluation alone. Nucleic acid amplification techniques (NAATs) have been increasingly recognized and implemented as the preferred method for identification of respiratory bacteria and viruses, including *M. pneumoniae*, in clinical specimens as a result of the high level of sensitivity and specificity and rapid turnaround time afforded by these methods. NAATs for *M. pneumoniae* were reviewed by Loens et al. (2003b), at which time only two of the 34 assays described for detection of *M. pneumoniae* were real-time PCR methods. Since that time, numerous real-time PCR assays encompassing a variety of chemistries have been developed and have largely replaced conventional PCR for research and diagnostic purposes. The predominant real-time PCR chemistries utilized for *M. pneumoniae* detection are intercalating dyes and 5′ hydrolysis (TaqMan^®^) assays. The most common genetic target regions within the *M. pneumoniae* genome are 16S rRNA, P1 gene, or the ATPase operon (Loens et al., 2003b). More recently, the gene encoding the community-acquired respiratory distress syndrome (CARDS) toxin, first described in Kannan et al. (2005), has also proven to be a useful target sequence for *M. pneumoniae* detection by real-time PCR (Winchell et al., 2008; Thurman et al., 2011).

A subsequent review of the status of *M. pneumoniae* diagnostics in 2010 detailed the rapid rise in laboratory-developed NAATs, specifically real-time PCR, multiplex or multi-pathogen PCR, and isothermal amplification methods, and emphasized the need to properly evaluate new assays prior to implementation (Loens et al., 2010). A lack of qualified standards used to evaluate new assays makes an accurate comparison of performance characteristics impossible. The external quality assessment (EQA) panel for *M. pneumoniae* available from Quality Control for Molecular Diagnostics (Glasgow, Scotland, United Kingdom) provides a useful metric for assessment of new laboratory-developed NAATs. Implementation of controlled standards for assessment of new methods will be beneficial for advancing *Mycoplasma* diagnostics. In addition to the numerous in-house laboratory-developed tests, several real-time PCR assays are now commercially available. A few recent studies have been conducted to evaluate the performance of these products, which overall were found to have comparable sensitivity and specificity albeit at a higher per specimen cost compared to LDTs (Dumke and Jacobs, 2009, 2014; Touati et al., 2009). Still, testing practices for detecting *M. pneumoniae* in the primary care setting are unlikely to change without modifications to guidelines for pathogen-specific testing established by relevant medical professional organizations, such as the IDSA. Periods of high incidence of *M. pneumoniae* infections, such as the recent worldwide epidemic period described in 2010–2012 (Lenglet et al., 2012; Diaz et al., 2015b; Kim et al., 2015), serve to bring *M. pneumoniae* to the attention of primary care providers and key decision-makers in the creation of guidelines for the treatment of CAP (Jacobs, 2012).

### Multiplex and Multi-Pathogen NAATs

Clinical presentation of *M. pneumoniae* infection can vary significantly and may be indistinguishable from respiratory infections caused by other bacterial and viral agents. Like *M. pneumoniae*, the *Chlamydophila* species are fastidious and extremely slow and difficult to culture. For these reasons, assays for detection of other atypical causes of bacterial pneumonia, including *Chlamydophila pneumoniae* or *C. psittaci*, as well as *Legionella* species, are often combined with *M. pneumoniae* into multiplex PCR formats (Miyashita et al., 2004; McDonough et al., 2005; Thurman et al., 2011). Diaz and Winchell (2012) described a rapid real-time PCR assay for detection of *M. pneumoniae* and *C. pneumoniae* that can be performed directly from clinical specimens without a nucleic acid extraction step about six times faster compared to standard real-time PCR methods. Although the sensitivity of the direct PCR was lower compared to extracted nucleic acid, it is possible that improved sensitivity could be achieved through further optimization of the method along with immediate testing of an upper respiratory swab at the time of collection, thus allowing for performance of this assay at the point-of-care. The requirement for a thermocycler instrument and procedural separation to avoid laboratory contamination and potential false positive results remain the most significant barriers to implementation of even the simplest and fastest real-time PCR methods in the clinical setting.

*Mycoplasma pneumoniae* has also been included in multi-pathogen panels for detection of a diverse array of bacterial, viral, and fungal agents capable of causing pneumonia, such as the BioFire FilmArray respiratory panel (BioFire Diagnostics, Salt Lake City, UT, USA), which is cleared for diagnostic use by the U.S. Food and Drug Administration (FDA; Poritz et al., 2011), and the Fast-track Diagnostics Respiratory Pathogens multiplex real-time RT-PCR assay kits (Fast-track Diagnostics, Luxembourg, Belgium) and Seegene Allplex Respiratory Full Panel Assay (Seegene, Inc., Seoul, Korea), which are CE certified in Europe. The U.S. Centers for Disease Control and Prevention (CDC) includes *M. pneumoniae* in a multi-pathogen testing panel on the TaqMan Array Card (TAC; ThermoFisher Scientific, CA, USA) used for investigating unexplained respiratory disease outbreaks in the United States (Kodani et al., 2011; Cieslak et al., 2012). Since 2011, TAC has been used by our laboratory for surveillance testing at the U.S. CDC to identify at least seven outbreaks of *M. pneumoniae* and was used to implicate *M. pneumoniae* as the cause of a cluster of severe CAP cases (Rhea et al., 2014; Waller et al., 2014; Diaz et al., 2015b; Hastings et al., 2015). Implementation of multi-pathogen detection methods could profoundly improve determination of incidence of infections caused by *M. pneumoniae* and impact appropriate antibiotic prescribing during CAP.

It is important to note that multiplex detection approaches for respiratory infections, including CAP, introduce additional complexity into the determination of etiology since the mere presence of an organism does not indicate a contribution to disease. Many bacterial and viral agents with pathogenic potential may also be present in the upper respiratory tract in a carriage state or for a prolonged period of shedding after resolution of infection in apparently healthy individuals (Hammitt et al., 2006; Roberts et al., 2012; Tenenbaum et al., 2012; Self et al., 2015; Skevaki et al., 2015). Frequent detection of *M. pneumoniae* in asymptomatic controls has been reported among children in the Netherlands (Spuesens et al., 2013), although this same phenomenon was not observed in a recent study of CAP etiology among children in the United States (Jain et al., 2015). Co-detections of other bacterial and viral pathogens along with *M. pneumoniae* have been reported in children (Michelow et al., 2004; Peng et al., 2009; Chiu et al., 2015). However, the significance of the presence of additional pathogens in the upper respiratory tract and their potential interplay with *M. pneumoniae* is not known. Expanded testing of respiratory specimens to include a wide collection of potential pathogens will present a challenge to clinicians for interpreting the true etiology of disease. Further investigation is needed to fully understand these interactions, and the movement of next generation sequencing techniques to clinical microbiology laboratories may help resolve some of these questions.

Real-time PCR has become a mainstream diagnostic procedure in reference laboratories and in some clinical laboratories. However, substantial barriers to implementation of this testing method in all clinical laboratories remain. Equipment for real-time PCR is expensive and requires routine preventative maintenance to ensure proper function. Laboratories need to have proper procedural separation of space and training of laboratory personnel, and all clinical laboratories offering patient testing in the United States must comply with the Clinical Laboratory Improvement Amendments (CLIA). Even when real-time PCR is available as a diagnostic test order, it is often not requested by physicians since current treatment guidelines recommend empiric antibiotic therapy without testing for suspected *M. pneumoniae* infection (Mandell et al., 2007; Bradley et al., 2011). As a result, significant effort has recently been invested in developing simpler NAATs or other detection approaches that may be utilized at the point-of-care, which will be summarized in the following sections.

### Isothermal Amplification Assays

Isothermal NAATs that do not require thermal cycling and are amenable to simple visual readout have also been developed for detection of *M. pneumoniae*, although these are less common than real-time PCR methods. Visualization of target amplification can be achieved using a fluorescent intercalating dye, chemiluminescent reporter, or even simple optical density (turbidity) measurement. These assays can be performed using minimal equipment, requiring only a standard heat block instead of a thermocycler with optical capability. Isothermal amplification assays have potential utility as point-of-care testing methods since they require only basic inexpensive equipment and minimal operator training, and they are relatively rapid. The two primary isothermal amplification methods that have been described for detection of *M. pneumoniae* are nucleic acid sequence-based amplification (NASBA) and loop-mediated isothermal amplification (LAMP).

#### Nucleic Acid Sequence-Based Amplification (NASBA)

NASBA is a unique method in that it is used for detection of RNA only through a process in which double stranded DNA (dsDNA) is generated from RNA through the use of avian myeloblastosis virus (AMV) reverse transcriptase (AMV-RT), T7 RNA polymerase, and RNase H while maintaining a constant reaction temperature of approximately 41°C. The dsDNA product is detected through an electrochemiluminescent readout for conventional assays or, more recently, through detection of fluorescent signal from a molecular beacon probe. Similar to PCR, real-time NASBA methods have largely replaced conventional NASBA assays. Loens et al. (2003a) described a real-time NASBA assay for detection of *M. pneumoniae* that performed comparably to a previously described conventional NASBA method in terms of analytical and clinical sensitivity (Loens et al., 2002). This assay was subsequently expanded to a multiplex format for simultaneous detection of *M. pneumoniae, C. pneumoniae*, and *Legionella* species (Loens et al., 2008).

Despite favorable assay performance characteristics and advantages of minimal equipment and operator expertise, NASBA has not been widely implemented in clinical laboratories to date. It may be that clinical laboratories have not yet updated test offerings from traditional methods such as serological analysis; alternatively, it is possible that laboratories prefer to offer real-time PCR for *M. pneumoniae*, for which there are numerous well-validated assays available and the technology is now widely accepted in diagnostic microbiology compared to NASBA. Procurement, inventory management, and quality control of additional specific reagents for NASBA testing for a single diagnostic test represents a significant investment of financial and personnel resources for a laboratory. Rather, it is more likely a NASBA assay could be implemented at the point-of-care than in clinical or reference laboratories, although substantial procedural optimization is required to make this feasible.

#### Loop-Mediated Isothermal Amplification (LAMP)

LAMP utilizes four to six primers and the strand-displacing DNA polymerase *Bst* to generate concatenated amplicons through a process in which stem-loop structures are introduced flanking the amplified target sequence. Several LAMP assays have been reported for the detection of *M. pneumoniae* (Saito et al., 2005; Yoshino et al., 2008; Gotoh et al., 2012; Petrone et al., 2015), and these vary in sensitivity and specificity due to both major and minor differences between the assays. Variables such as genetic target, reaction composition, and readout mechanism can significantly impact assay performance. Due to the complexity of the amplification events and high number of oligonucleotides, extensive optimization and validation is required to ensure reliability of results (Petrone et al., 2015).

The *illumigene* Mycoplasma DNA amplification assay (Meridien Bioscience, Inc., Cincinnati, OH, USA) is the only standalone assay to be cleared by the U.S. FDA for detection of *M. pneumoniae* in clinical specimens. This assay, which targets the intracellular protease gene, displayed 100% sensitivity and 99% specificity compared to culture (Ratliff et al., 2014). Still, the requirement for extraction of nucleic acid from the primary specimen hinders the implementation of this assay for point-of-care diagnostic use. Petrone et al. (2015) demonstrated successful detection of *M. pneumoniae* in clinical specimens directly without a nucleic acid extraction step using a novel LAMP assay targeting the CARDS toxin gene. The sensitivity of this LAMP assay for detection of *M. pneumoniae* using primary specimen in place of extracted nucleic acid was 82% compared to real-time PCR. This assay was optimized to utilize calcein as a fluorescent readout and can be performed in approximately 1 h from time of specimen collection to results. Improved sensitivity would be expected with appropriate modification of specimen collection procedures and immediate testing of specimens after collection. Further optimization is needed to formulate a simple user-friendly reaction setup in order for this method to be feasible for point-of-care testing.

## Detection of *M. pneumoniae* Using Antigen Detection Methods

Some detection methods rely on capture and detection of *M. pneumoniae*-specific antigen, rather than nucleic acid, present in clinical specimens. However, antigen detection methods such as enzyme-linked immunosorbent assay (ELISA) and hybridization assays have been largely replaced by NAATs due to the improved sensitivity, specificity, and rapid turnaround time. A complete review of these methods is outside of the scope of this review. Nonetheless, there remains some interest in antigen detection methods for diagnosis of *M. pneumoniae*, particularly those that may be amenable to point-of-care use. In addition to being sensitive and specific, point-of-care diagnostic tests must be extremely rapid (on the order of minutes), simple to perform, require little to no equipment, and be relatively inexpensive.

### Immunochromatographic (Lateral Flow) Assays

Immunochromatographic assays, also known as lateral flow assays, represent one of the simplest test formats available and have been widely implemented for various purposes, including pathogen detection, at the point-of-care (Posthuma-Trumpie et al., 2009). Several immunochromatographic assays for detection of *M. pneumoniae* antigen are currently available in Japan. These tests provide results within 15 min and are easily interpreted by visual observation of the presence or absence of a colored line on the test strip. Diagnosis and appropriate treatment of *M. pneumoniae* is particularly important in Asia where the majority of strains are resistant to macrolides (Cao et al., 2010; Okada et al., 2012), the recommended first-line antimicrobial therapy, as described in further detail in section “Macrolide Susceptibility Genotyping of *M. pneumoniae*” below.

Ribotest Mycoplasma^TM^ (Asahi Kasei Corporation, Tokyo, Japan), is one commercially available rapid diagnostic lateral flow assay for qualitative detection of the ribosomal protein L7/L12 of *M. pneumoniae* in pharyngeal swab specimens. However, Miyashita et al. (2015) recently reported a diagnostic sensitivity of only 60% for Ribotest^TM^ compared to real-time PCR, indicating that this assay does not meet the level of sensitivity required for detection of *M. pneumoniae* in clinical specimens. Recently, Li et al. (2015) reported a novel colloidal gold immunochromatographic antigen assay for detection of the P1 gene of *M. pneumoniae* that was 100% sensitive and 97.4% specific compared to real-time PCR. A highly sensitive and specific assay with broad market distribution will be required to achieve widespread implementation in clinical laboratories.

### Nanorod Array Surface-Enhanced Raman Spectroscopy (NA-SERS)

Another alternative approach that has been proposed for detection of *M. pneumoniae* is nanorod array surface-enhanced Raman spectroscopy (NA-SERS). This method involves generation of a metallic nanorod array substrate and application of Raman spectroscopy to detect the unique vibrational spectral profile of biomolecules in an applied sample. Hennigan et al. (2010) demonstrated the ability of NA-SERS to detect *M. pneumoniae* in mock or true clinical specimens with sensitivity comparable to real-time PCR methods. Further evaluation revealed the ability of this method to differentiate *M. pneumoniae* from other commensal and pathogenic *Mycoplasma* species and to further differentiate strain types (Henderson et al., 2015), as described further in section “Nanorod Array Surface Enhanced Raman Spectroscopy” below. While this method provides a unique and promising alternative strategy for *M. pneumoniae* detection, significant challenges remain for implementation in clinical laboratories, including required equipment, specimen processing, and application of statistical analysis to evaluate the spectral profile identified in clinical specimens. Furthermore, substantial testing will be necessary to evaluate the feasibility of this approach for *M. pneumoniae* detection in the presence of co-detected pathogens and normal microbial flora of the upper respiratory tract.

### Mass Spectrometry

Matrix-assisted laser desorption ionization-time-of-flight mass spectrometry (MALDI-TOF MS) is a useful technique for the rapid identification of pathogenic microorganisms, including both Gram-positive and Gram-negative bacteria, based on the unique spectral profile of proteins in bacterial lysate. Pereyre et al. (2013) generated peptide mass fingerprint product ion spectra for 10 human and 13 ruminant *Mycoplasma* species or subspecies in order to develop a main spectra (MSP) database for identification of clinically relevant *Mycoplasma* species, including *M. pneumoniae*. The dendrogram based on 29 MSPs from 23 mycoplasmas was consistent with 16S rRNA phylogeny (Pereyre et al., 2013). This method was sufficiently sensitive to discriminate closely related *Mycoplasma* species, but is limited by the requirement for a culture isolate, which may take weeks to obtain due to the slow growth of *M. pneumoniae*. Furthermore, a large volume of culture (30–100 mL) was required for extraction of proteins for successful generation of MSPs; the time required and means to generate such a high volume culture are not practical in most clinical laboratories. Nonetheless, MALDI-TOF MS has been shown to be useful for the detection of anaerobic, fastidious, and slow-growing bacterial isolates from clinical specimens (Biswas and Rolain, 2013). In order for MALDI-TOF MS to meet the rapid turnaround time possible with NAATs, technical optimization is needed to achieve detection of *M. pneumoniae* directly from respiratory specimens.

## Characterization of *M. pneumoniae*

*Mycoplasma pneumoniae* is a highly genetically conserved species; genomic comparisons have revealed >99% sequence similarity between isolates (Lluch-Senar et al., 2015; Xiao et al., 2015). Still, several methods have been developed to characterize *M. pneumoniae* strains based on various genetic elements and allowing for classification of *M. pneumoniae* for epidemiological purposes. In the absence of substantial sequence diversity, strain differentiation efforts have focused largely on variability in the relatively high number of repetitive elements within the genome. Approximately 8% of the *M. pneumoniae* genome is comprised of repetitive sequences, some of which are present in multiple copies throughout the genome (Himmelreich, 1996). Variation in the nucleotide sequence or the number of tandem repeats at these genetic loci underlie some of the most commonly used methods for *M. pneumoniae* typing.

Recent advances in WGS and access to an increasing collection of publicly available complete *M. pneumoniae* genomes (discussed in depth in section “Whole Genome Sequencing” below) have aided in identifying areas within the genome that can be targeted to achieve greater discriminatory power. With the expansion of WGS to the clinical sector, there is little doubt that genomic characterization of *M. pneumoniae* will become more reliable and robust, and WGS analysis may be routinely used during outbreak investigations or as part of surveillance programs. In the following sections, we review the current approaches for typing of *M. pneumoniae* and describe the methods for each characterization scheme, culminating in a review of recent WGS advancements and a discussion of the future application of WGS to *M. pneumoniae* diagnostics.

### P1 Typing

Typing based on sequence variation within repetitive elements located in the gene encoding the P1 adhesion molecule was first described in 1990 and has the longest history of use for distinguishing the two main subtypes of *M. pneumoniae*, types 1 and 2 (Dallo et al., 1990; Su et al., 1990b). Two of the repetitive elements found in the *M. pneumoniae* genome, RepMP2/3 and RepMP4, are located within the gene encoding the 170 kDa adhesin protein P1, and sequence variation between types 1 and 2 strains occurs largely within these repetitive regions (Su et al., 1990a). In addition, 7 copies of RepMP4 and nine copies of RepMP2/3 have been identified at various loci in the genome outside of the transcriptionally active operon that includes the P1 gene (Ruland et al., 1990; Himmelreich, 1996). Evidence of recombination of these sequence copies into the transcribed P1 gene has been reported, yet the exact mechanisms underlying these recombination events and the frequency of such events are not known (Spuesens et al., 2009; Musatovova et al., 2012). Notably, Spuesens et al. (2009) found that isolates contain either types 1 or 2-specific RepMP sequences within their genome, but not both, suggesting an early divergence in the phylogeny of *M. pneumoniae*. Variants of each type have also been described (Schwartz et al., 2009a; Spuesens et al., 2009; Zhao et al., 2011; Kenri et al., 2012).

The P1 adhesin is a major virulence determinant of *M. pneumoniae*, facilitating adherence of the bacteria to respiratory epithelial cells during infection (Baseman et al., 1996; Baseman and Tully, 1997; Razin et al., 1998; Waites and Talkington, 2004; Atkinson et al., 2008). P1 is a primary immunogenic component of *M. pneumoniae*, and thus sequence variation within the P1 gene can be expected to result in alteration in the surface-exposed protein thereby potentially affecting the infectious process. In fact, the alternating predominance of types 1 or 2 strains circulating in a population during epidemic seasons has been documented previously (Lind et al., 1997; Kenri et al., 2008; Kogoj et al., 2015), and the cyclic pattern was potentially attributed to the development of temporary immunity to one type, thus allowing reemergence of the other type (Dumke et al., 2004). However, recently co-circulation of both P1 types and multiple variants have been reported during the same epidemic period and even during discrete outbreaks (Waller et al., 2014; Diaz et al., 2015b; Jacobs et al., 2015). These findings suggest that P1 typing alone is likely not adequate to classify *M. pneumoniae*.

Furthermore, the lack of any association of strain type with disease characteristics, particularly severity of illness or patient outcomes, calls into question the utility of P1 typing and other typing schemes included in this review. However, there remains continued interest and benefit in monitoring strain types using existing methods in order to understand the epidemiological shifts in circulating *M. pneumoniae* strains over time and across geographic locations. Newer methods have been developed with superior discriminatory power compared to P1 typing alone, yet, even using WGS analysis, the most comprehensive characterization method available, strains are still classified into 2 main clades corresponding to P1 types, although further separation within these clades has been observed using WGS (Lluch-Senar et al., 2015). Although P1 typing is likely to be augmented or replaced by newer methods to better characterize *M. pneumoniae*, it will be used for some time still for epidemiological investigations and surveillance programs. Here we review the primary methods used for typing *M. pneumoniae* based on the P1 adhesin (**Table 1**).

**Table 1:** Molecular methods for characterization of *Mycoplasma pneumoniae.*

Characterization scheme	Method(s)	Reference(s)
P1 gene typing	PCR-RFLP	Cousin-Allery et al., 2000
	PCR-high-resolution melt (HRM)	Schwartz et al., 2009b
	NASBA	Ovyn et al., 1996
	Sequencing	Dumke et al., 2006
	Pyrosequencing	Spuesens et al., 2010
	MALDI-TOF MS	Xiao et al., 2014
	NA-SERS	Henderson et al., 2015
Multilocus Variable-Number Tandem-Repeat Analysis (MLVA)	MLVA	Degrange et al., 2009
	MLVA (nested PCR)	Dumke and Jacobs, 2011
Multilocus sequence typing (MLST)	MLST	Brown et al., 2015b
Single nucleotide polymorphism (SNP) genotyping	SNaPshot minisequencing assay	Touati et al., 2015
Macrolide susceptibility genotyping	Sequencing	Lucier et al., 1995
	Pyrosequencing	Spuesens et al., 2010; Chan et al., 2013
	PCR-melting curve analysis	Wolff et al., 2008; Chan et al., 2013
Whole genome sequencing (isolates)	Shotgun sequencing	Himmelreich et al., 1996
	High-throughput sequencing	Lluch-Senar et al., 2015; Xiao et al., 2015
	Single-molecule long-read sequencing	Lluch-Senar et al., 2013

#### Restriction Fragment Length Polymorphism (RFLP) and Sequencing Analysis

One widely used approach to typing *M. pneumoniae* based on P1 is PCR-Restriction Fragment Length Polymorphism (PCR-RFLP) analysis (Sasaki et al., 1996; Cousin-Allery et al., 2000; Kenri et al., 2008; Musatovova et al., 2008). Results are compared to prototypical type strains of each type, M129 (type 1) and FH (type 2). Using this method, *M. pneumoniae* isolates can be identified as type 1 or 2 or a number of unique variants. Dumke et al. (2006) utilized amplification and sequencing to distinguish P1 types directly from clinical specimens. Spuesens et al. (2010) reported the use of pyrosequencing for molecular typing of *M. pneumoniae* into the two main subtypes based on sequence variation in the MPN141 (P1) and MPN528a genes. Both of these methods require post-PCR processing, which increase time to results and potential for contamination of laboratory space with PCR amplicon, which may lead to false positive results. This risk can be mitigated by meticulous separation of space and equipment for post-PCR processing steps.

#### High Resolution Melt (HRM) Analysis

Schwartz et al. (2009a,b) reported a novel PCR assay with High Resolution Melt (HRM) analysis to differentiate types 1 and 2 and to identify variants. This approach uses amplification of a 1900 bp sequence followed by melting curve analysis in a one-step reaction to clearly distinguish types 1 and 2 isolates based on alteration in the melting temperature created by multiple single nucleotide polymorphisms (SNPs) located within the amplicon. Variants of type 1 or 2 are also identifiable by virtue of further sequence variation in the amplified target region (Schwartz et al., 2009a). The major advantages of this method are a more rapid turnaround time and no requirement for post-PCR reaction manipulation, dramatically reducing the risk of amplicon contamination in the laboratory.

#### Nucleic Acid Sequence-Based Amplification (NASBA)

In addition to typing based on analysis of the P1 gene, it has been reported that there is one SNP in the 16S rRNA gene that can be used to differentiate types 1 and 2 strains. Ovyn et al. (1996) developed a conventional NASBA assay which allows differentiation of the two main P1 types based upon binding of the electrochemiluminescent-labeled hybridization probe in the region containing this SNP. This SNP could be easily detected using targeted resequencing methods or identified in whole genome sequences in order to simply and reliably identify the main P1 types.

### Multi-Locus Variable Number Tandem Repeat (VNTR) Analysis (MLVA)

MLVA is a technique applied to many bacterial species for strain differentiation based upon the number of tandemly repeated sequences located at designated loci throughout the genome. Approximately 8% of the *M. pneumoniae* genome is comprised of repetitive elements (Himmelreich, 1996), making this species well-suited for characterization using MLVA. In 2009, Degrange et al. (2009) developed a five-loci MLVA scheme for differentiation of *M. pneumoniae* strains through identification and selection of VNTR regions in *M. pneumoniae* that were polymorphic between isolates, yet stable upon passage in broth culture. Using this five-loci typing scheme, 26 MLVA types were identified, and these were assigned alphabetical identifiers, A through Z. Shortly after the development of this method, a slightly modified protocol was applied which enabled testing of nucleic acid extracted from primary clinical specimens, thus eliminating the need for a culture isolate (Dumke and Jacobs, 2011; Benitez et al., 2012). Since the introduction of this method for *M. pneumoniae* characterization, it has been widely implemented for investigating outbreaks as well as characterizing historical strain collections. However, in the course of implementing this method, the first locus, Mpn1, was shown to be unstable, rendering it impractical for classification of *M. pneumoniae* (Benitez et al., 2012; Sun et al., 2013). Multiple research groups proposed the exclusion of this marker and modification to a four-loci MLVA scheme (Sun et al., 2013; Waller et al., 2014). The removal of the Mpn1 locus from the typing scheme reduced the discriminatory power of this method, allowing classification of *M. pneumoniae* strains into fewer unique types. Nonetheless, the modified approach is generally considered to be more robust and has been accepted as the new international standard for *M. pneumoniae* MLVA typing (Chalker et al., 2015).

Adaptation to the four-loci MLVA typing scheme revealed a few predominant MLVA types circulating concurrently in the past 5–7 years, a period during which increased *M. pneumoniae* cases were documented on multiple continents, including Europe, Asia, and North America (Chalker et al., 2011, 2012; Blystad et al., 2012; Eibach et al., 2012; Lenglet et al., 2012; Nir-Paz et al., 2012; Polkowska et al., 2012; Eshaghi et al., 2013; Sun et al., 2013; Diaz et al., 2015b). The three most common types identified during this period were 4572, 3562, and 3662 (Sun et al., 2013; Diaz et al., 2015b). Adaptation to the four-loci MLVA typing scheme in our laboratory revealed a correlation between MLVA type and P1 type; isolates identified as MLVA type 4572 were always P1 type 1 while MLVA type 3562 or 3662 were always P1 type 2 (Waller et al., 2014; Diaz et al., 2015b). While this correlation has held up for all isolates and specimens tested to date in our laboratory, other investigators have reported a small number of P1 type 1 strains that are not MLVA type 4572 and P1 type 2 strains that are not MLVA type 3X62, suggesting that there are few exceptions to this correlation (Degrange et al., 2009; Dumke and Jacobs, 2011; Sun et al., 2013). Nonetheless, the biological reasons underlying the observed correlation are not well understood, particularly since these four VNTR regions are located either within an intergenic region (Mpn13) or in an open reading frame (ORF) encoding a hypothetical protein [Mpn14 (MPN 501), Mpn15 (MP 524), and Mpn16 (MPN 613)] (Degrange et al., 2009). However, recent whole genome SNP and indel analysis of numerous *M. pneumoniae* isolates also substantiates the separation of these two main groups (Lluch-Senar et al., 2015; Xiao et al., 2015), supporting the early phylogenetic divergence of two main lineages of *M. pneumoniae* (Musatovova et al., 2012).

While MLVA has higher discriminatory power compared to P1 typing (Pereyre et al., 2012), researchers have continued to pursue the development of typing methods with even higher discriminatory power or that are clinically or epidemiologically informative. Some recent reports have suggested a correlation of MLVA type 4572 with macrolide resistance and disease severity (Qu et al., 2013; Ho et al., 2015). Other studies have reported an association between macrolide resistance and disease severity or clinical course (Cardinale et al., 2013; Zhou et al., 2014); thus, additional studies are needed to determine strain attributes that may impact the course of *M. pneumoniae* infection. Further investigation is necessary to verify any potential associations since these findings may influence testing practices for *M. pneumoniae* in clinical laboratories and could ultimately improve patient management.

### Multi-Locus Sequence Typing (MLST)

MLST is a widely used tool for strain differentiation in many genera of bacteria. Initial attempts to categorize *M. pneumoniae* isolates into MLST types using housekeeping and structural genes were generally unsuccessful due to limited sequence variation within these regions (Dumke et al., 2003). Using a growing set of whole genome sequence data, a new MLST method was recently reported by Brown et al. (2015b) that exploited sequence polymorphisms of eight housekeeping genes (*ppa, pgm, gyrB, gmk, glyA, atpA, arcC*, and *adk*). SNPs were identified in the type strains of *M. pneumoniae* (M129 and FH) and 35 clinical isolates. Further sequencing and PCR experimentation with an additional 20 isolates allowed for 12 distinct sequence types (STs) to be established. This is substantially more discriminating than the previous MLST scheme that only found slight sequence variation in the three housekeeping genes selected for discrimination (Dumke et al., 2003). The authors also confirmed the relative stability of these MLST loci after performing 10 sequential subculture passages of isolates, finding no change in the SNP patterns. Like other typing methods, no link between the reported STs and isolation year, patient age, or geographic origin of the clinical specimen, was found. However, two distinct genetic clusters were observed that correlate with MLVA type 4572 and 3X62. The two clonal complexes resulting from this more comprehensive MLST study underscore the significant differences between these two genetically distinct lineages.

Although this typing scheme is more discriminating than the commonly used MLVA and P1 typing methods, it still requires PCR and sequencing of the isolate to generate the ST. Greater utility of this procedure will surely be realized when this methodology is applicable for testing directly on clinical specimens. Nonetheless, this method is of value for typing isolates for epidemiological investigations and can greatly enhance the understanding of strain circulation, transmission dynamics, and relative persistence in a population or geographic location. The creators of this scheme also established a web-based database for *M. pneumoniae* MLST data that can also be linked to an isolate database that contains epidemiological information (Jolley and Maiden, 2010). New data can be submitted to the database in order to track the number of unique profiles identified to date.

### Single Nucleotide Polymorphism (SNP) Genotyping

Another molecular typing approach used to more clearly define and genotype *M. pneumoniae* isolates and positive clinical specimens is based upon SNPs that were identified after performing WGS on eight strains. Touati et al. (2015) showed that nine SNP types can be determined from eight reliable SNPs identified within housekeeping, predicted lipoprotein, and P1 adhesin genes using a “SNaPshot” mini-sequencing assay. This approach uses a single-base extension (SBE) method which allows an unlabeled mini sequencing primer to anneal one base upstream of the specified SNP using a fluorescently labeled ddNTP that can be easily detected after the separation and extension of the product has occurred. This technology was used to characterize 140 *M. pneumoniae* strains previously typed using five-loci MLVA and P1 methods. These previously used typing schemes had mixed correlation when compared to the SNaPshot mini-sequencing procedure; SNP genotyping correlated poorly with 5-loci MLVA types but strongly with P1 types (Touati et al., 2015). The poor correlation with the 5-loci MLVA type may be a result of the instability of the first locus, Mpn1, which introduces artefactual differences between strains. The SNP typing method had a higher Hunter and Gaston diversity index compared to other typing methods, including 4-loci MLVA. Other major advantages of this technology are that it can be highly multiplexed, has increased sensitivity, and can be used directly on clinical specimens (Sobrino et al., 2005; Touati et al., 2015). Identification of SNPs in nucleic acid from clinical specimens without performing any sequencing procedure affords a significant savings in time and cost while also mitigating any potential contamination within laboratories since minimal manipulation of the specimen is required. Furthermore, this method allows for discrimination of strains without the need to generate and handle WGS data, which requires substantial computing power and bioinformatics expertise. This SNP typing method may prove to be useful for epidemiological analysis, but is likely to only be performed at highly specialized academic and reference laboratories.

### MALDI-TOF MS + ClinProTools

Initial evaluation of MALDI-TOF MS method for identification of *M. pneumoniae* (described in section “Identification of *M. pneumoniae* Using Mass Spectrometry” above) also revealed that strains clustered by P1 type based upon the MSP (Pereyre et al., 2013). Xiao et al. (2014) utilized an analysis software tool, ClinProTools (Bruker Daltonics, Bremen, Germany) to differentiate P1 types using a genetic algorithm based upon seven biomarker peaks identified using MALDI-TOF MS. These investigators demonstrated that the genetic algorithm model was able to correctly identify the P1 type of 43 *M. pneumoniae* isolates based upon the peptide mass fingerprints. Thus, P1 typing can be successfully performed either by analysis of the nucleic acid or protein composition of an isolate. To date, nucleic acid-based approaches have been preferred methods by most laboratories, but MALDI-TOF MS is rapidly gaining acceptance in the clinical microbiology field and thus represents a potential future avenue for *M. pneumoniae* diagnostics.

### Nanorod Array Surface Enhanced Raman Spectroscopy (NA-SERS)

The NA-SERS method described in section “Nanorod Array Surface-Enhanced Raman Spectroscopy” was also capable of differentiating *M. pneumoniae* strains into three classes corresponding to P1 types 1, 2 and 2 variant (2V) (Henderson et al., 2015). Partial least squares-discriminatory analysis (PLS-DA) modeling was applied to differentiate *M. pneumoniae*-specific spectra from background and the spectra of other pathogenic or commensal mycoplasmas. Subsequently, three unique PLS-DA models were built to distinguish strains based on P1 type. Although this method does not provide a higher discriminatory power compared to other typing methods, it does allow for simultaneous detection and typing of *M. pneumoniae* in a rapid and reliable manner. However, unless a clinically relevant association of strain type with disease is identified, performance of typing assays in a clinical laboratory is of little value for patient care, and typing methods are likely to remain an offering only by specialized reference laboratories. In addition, further validation is needed to assess performance of this method for detection and typing of *M. pneumoniae* strains in complex clinical specimens. Nonetheless, NA-SERS represents a unique alternative approach to the identification of *M. pneumoniae* and other respiratory pathogens, particularly if this method can be modified for multi-pathogen detection in clinical specimens.

## Macrolide Susceptibility Genotyping of *M. pneumoniae*

Macrolides, primarily azithromycin, are the recommended first-line antibiotic for treatment of *M. pneumoniae* (Waites and Talkington, 2004; Mandell et al., 2007; Atkinson et al., 2008; Bradley et al., 2011; Waites, 2011). Since the first report in 2001 of macrolide-resistant *M. pneumoniae* (Okazaki et al., 2001), the prevalence of this trait has emerged worldwide, reaching dangerously high levels upward of 90% in Asia (Liu et al., 2009; Xin et al., 2009; Cao et al., 2010; Okada et al., 2012). In the United States and Europe, macrolide resistance has persisted over the past decade, albeit at relatively low levels (∼10%; Steffens et al., 2012; Diaz et al., 2015a,b; Zheng et al., 2015). However, diagnostic testing for detection of *M. pneumoniae* is not routinely performed in the United States, so specimens are not often available for susceptibility testing. Estimates of the prevalence of macrolide resistance in the United States are generally obtained from outbreak investigations and limited surveillance studies. Three recent studies have reported macrolide resistance rates ranging from 3.5 to 13.2% among *M. pneumoniae*-positive clinical specimens in the United States (Diaz et al., 2015a,b; Zheng et al., 2015). The highest rate reported in the United States, 27%, occurred during a discrete outbreak in Rhode Island in 2009, although this was based on only 11 specimens (Wolff et al., 2008). Resistance rates from large-scale multi-site surveillance studies likely represent a more accurate estimate, but it remains possible that macrolide resistance is more likely to develop during prolonged outbreaks.

Some studies suggest that resistance develops in an individual patient in response to macrolide treatment (Dumke et al., 2014), although the frequency with which this occurs is not well defined and may be low (Nilsson et al., 2014). Development and expansion of a resistant subpopulation within an individual patient in response to macrolide therapy rather than transmission of a resistant isolate within a population is supported by investigations of outbreaks and transmission among household contacts in which only a few sporadic clinical isolates were found to be resistant (Diaz et al., 2015b). Still, the rapid emergence of macrolide resistant *M. pneumoniae* in Asia compared with the relatively low and stable presence of these strains in Europe and North America underscore that this trait must be studied at the population level rather than only within an individual patient. Further investigation including longitudinal studies are needed in order to understand how the resistance trait emerges and expands within a population.

The mechanism of resistance to macrolides in *M. pneumoniae* is well understood; a single SNP at one of several key residues within or adjacent to the binding site in the peptidyl transferase loop of the 23S rRNA large subunit prevents the macrolide from binding and inhibiting protein synthesis (Bebear et al., 2011). Mutations at positions 2063 and 2064 in *M. pneumoniae* result in high level resistance to macrolide antibiotics, whereas a mutation at position 2067 or 2617 is associated with a lower level of resistance (Morozumi et al., 2010). Mutations that occur in the 23S rRNA gene are dominant as there is only a single rRNA operon in the *M. pneumoniae* genome (Gobel et al., 1984). Since only a single base change confers resistance, it is biologically plausible that this event may happen frequently, especially since *M. pneumoniae* is known to have limited DNA repair mechanisms in the reduced genome (Carvalho et al., 2005). Furthermore, the relatively long biological half-life of macrolide antibiotics, particularly azithromycin, may also contribute to the development of resistance *in vivo* (Stevens et al., 1997; Kastner and Guggenbichler, 2001).

Molecular methods have been developed using a variety of techniques to rapidly determine susceptibility of a *M. pneumoniae* isolate or primary clinical specimen extract. While these methods vary in complexity, all require a substantial investment of equipment, laboratory space, and highly trained staff for performance and, therefore, are generally restricted to reference and research laboratories. However, some studies indicate that infection with macrolide-resistant *M. pneumoniae* may be of longer duration or severity (Cardinale et al., 2013; Zhou et al., 2014), supporting the value of macrolide susceptibility testing for informing patient management, particularly in severe cases.

### Sanger Sequencing

Perhaps the most straightforward approach to identifying sequence polymorphisms in the 23S rRNA gene is to amplify the target region by conventional PCR and perform nucleotide sequencing analysis. Lucier et al. (1995) performed broth dilution tests, ribosomal binding studies, and DNA sequencing analysis to identify SNPs within 23S rRNA gene of *M. pneumoniae* that confer resistance to macrolide antibiotics. Subsequent studies have used Sanger sequencing as a comparative method to validate novel molecular assays for detection of known polymorphisms (Matsuoka et al., 2004; Wolff et al., 2008; Chan et al., 2013).

Recently, Dumke et al. (2014) reported the emergence of a macrolide-resistant subpopulation of *M. pneumoniae* within an individual patient by collection and testing of multiple specimens during the course of the infection. In this report, the investigators cloned PCR amplicons of 23S rRNA into a plasmid, selected colonies, and performed sequencing to identify the genotype present in the specimen. Using this method, only sensitive *M. pneumoniae* were detectable in the specimen collected on day 1, but a mixture of both sensitive and resistant sequences were detected in a specimen collected 18 days later (Dumke et al., 2014). Interestingly, resistant quasispecies containing either the A2063G or A2064G mutations (46 and 28%, respectively) were identified in the same specimen, along with the wildtype genotype (26%). While this method may be used to identify mixed populations of macrolide-sensitive and –resistant *M. pneumoniae* in a patient specimen, it is prohibitively cumbersome and time-consuming to be feasible for clinical testing or even as a routine procedure in specialty reference laboratories.

### Pyrosequencing

Pyrosequencing has been used in several studies to evaluate macrolide susceptibility of *M. pneumoniae* isolates or primary specimen extracts (Cao et al., 2010; Spuesens et al., 2010, 2012). For pyrosequencing, PCR is performed using a set of oligonucleotide primers, one of which has a biotin label. The resulting biotinylated PCR product is purified using streptavidin-coated beads, denatured, and subjected to sequencing. Pyrosequencing is the only method developed to date that is capable of quantifying the proportions of macrolide-sensitive and -resistant quasispecies within a clinical specimen (Chan et al., 2013) and is a more feasible approach for determination of mixed genotypes in clinical specimens compared to the cloning and sequencing method described in section “Sanger Sequencing.” Using pyrosequencing, Chan et al. (2013) determined that nearly 80% of *M. pneumoniae*-positive clinical specimens contained some proportion of macrolide-resistant quasispecies. In specimens that were previously identified as having the wildtype (macrolide-susceptible) genotype using another method, up to 44% of the *M. pneumoniae* population was found to be macrolide-resistant. Among specimens identified as macrolide-resistant by other methods, pyrosequencing revealed that the resistant quasispecies comprised 52–100% of the total population. These results underscore the potential for development of macrolide resistance during the course of infection in an individual patient. Longitudinal studies in which multiple specimens are collected from the same patient throughout the duration of illness will be necessary to demonstrate the emergence of macrolide-resistant *M. pneumoniae* resulting from macrolide therapy at an individual level. Adoption of methods capable of identifying quasispecies within a patient specimen, including pyrosequencing and potentially digital droplet PCR, could help monitor the emergence of resistance in this organism or to identify infections that are less likely to be resolved by macrolide therapy.

### Restriction Fragment Length Polymorphism (RFLP) Analysis

Matsuoka et al. (2004) established RFLP methods for analysis of point mutations in 23S rRNA in *M. pneumoniae*. RFLP has traditionally been used for typing of *M. pneumoniae* based on the P1 adhesion molecule as described in section “Restriction Fragment Length Polymorphism (RFLP) and Sequencing Analysis” above. Digestion of a 210 bp PCR product amplified from 23S rRNA with either BceAI or BsaI results in multiple fragments when either the A2063G or A2064G mutation is present compared to a single uncut fragment for amplified product containing the wildtype genotype (Matsuoka et al., 2004). While this method is reliable, it is not well-suited for use in clinical microbiology laboratories.

### Melting Curve Analysis

Wolff et al. (2008) described a PCR assay using high-resolution melt (HRM) analysis to rapidly differentiate macrolide-resistant and –susceptible isolates. Two versions of the assay were developed, using a specific primer set with an intercalating dye or a self-quenched fluorogenic LUX primer. The substitution of G for A at position 2063 or 2064 causes the amplicon to melt at a slightly higher temperature, thus the melting profile can reliably distinguish macrolide-resistant isolates by comparison to sensitive and resistant controls included in the run. However, this method does not identify the exact mutation present within the amplicon. Subsequently, the HRM assay was modified to include a nested PCR step, allowing for testing of nucleic acid from primary clinical specimens (Diaz et al., 2015b). Eliminating the need to obtain an isolate allowed the assay to be performed in sufficient time to inform patient treatment decisions.

Similarly, Chan et al. (2013) developed a SimpleProbe real-time PCR assay with melting curve analysis for detection of SNPs in 23S rRNA of *M. pneumoniae*. The SimpleProbe format consists of a single-labeled hybridization probe that emits higher fluorescence when bound to the specific target sequence containing the SNP of interest compared to emission in the unhybridized state. Binding of the probe to the PCR product that contains the SNP is less stable, causing it to melt at a lower temperature. This reaction can be performed with relatively rapid cycling conditions resulting in a turnaround time under 1 h (Chan et al., 2013), which represents a substantially faster time to results compared to other methods for macrolide susceptibility determination.

Recently, Nummi et al. (2015) reported the development of a multiplex real-time PCR assay for simultaneous detection of *M. pneumoniae, C. pneumoniae*, and the two most common mutations that confer macrolide resistance in *M. pneumoniae*. This method utilizes post-PCR dissociation curve analysis to identify macrolide-resistant 23S rRNA sequences amplified from clinical specimens. This type of assay, which provides simultaneous identification of *M. pneumoniae* and determination of macrolide susceptibility in patient specimens, would improve appropriate antibiotic prescribing for respiratory infections caused by this pathogen. Implementation of this type of method at the point-of-care would provide the best opportunity to impact prescribing and patient management. On a population level, widespread implementation of methods like this in surveillance programs would improve monitoring of macrolide resistance patterns, particularly as these may change rapidly and vary substantially based on geography.

## Whole Genome Sequencing (WGS)

The reduced genome makes *M. pneumoniae* amenable to high throughput WGS and other “omics” analyses. Vast improvements in WGS over the past decade have made this a more accessible approach for identification and characterization of bacteria. Sequencing platforms have evolved from whole genome shotgun sequencing (Sanger) to high-throughput sequencing (Roche 454 and Illumina) and finally to single-molecule long-read sequencing (PacBio SMRT sequencing and Oxford Nanopore sequencing; Loman and Pallen, 2015). The availability of benchtop sequencers has expanded WGS capacity in academic, clinical, and public health laboratories. This expansion in sequencing capability has resulted in a rapid increase in the number of bacterial genomes, including *M. pneumoniae*, made publicly available in the last several years.

**FIGURE 1:**
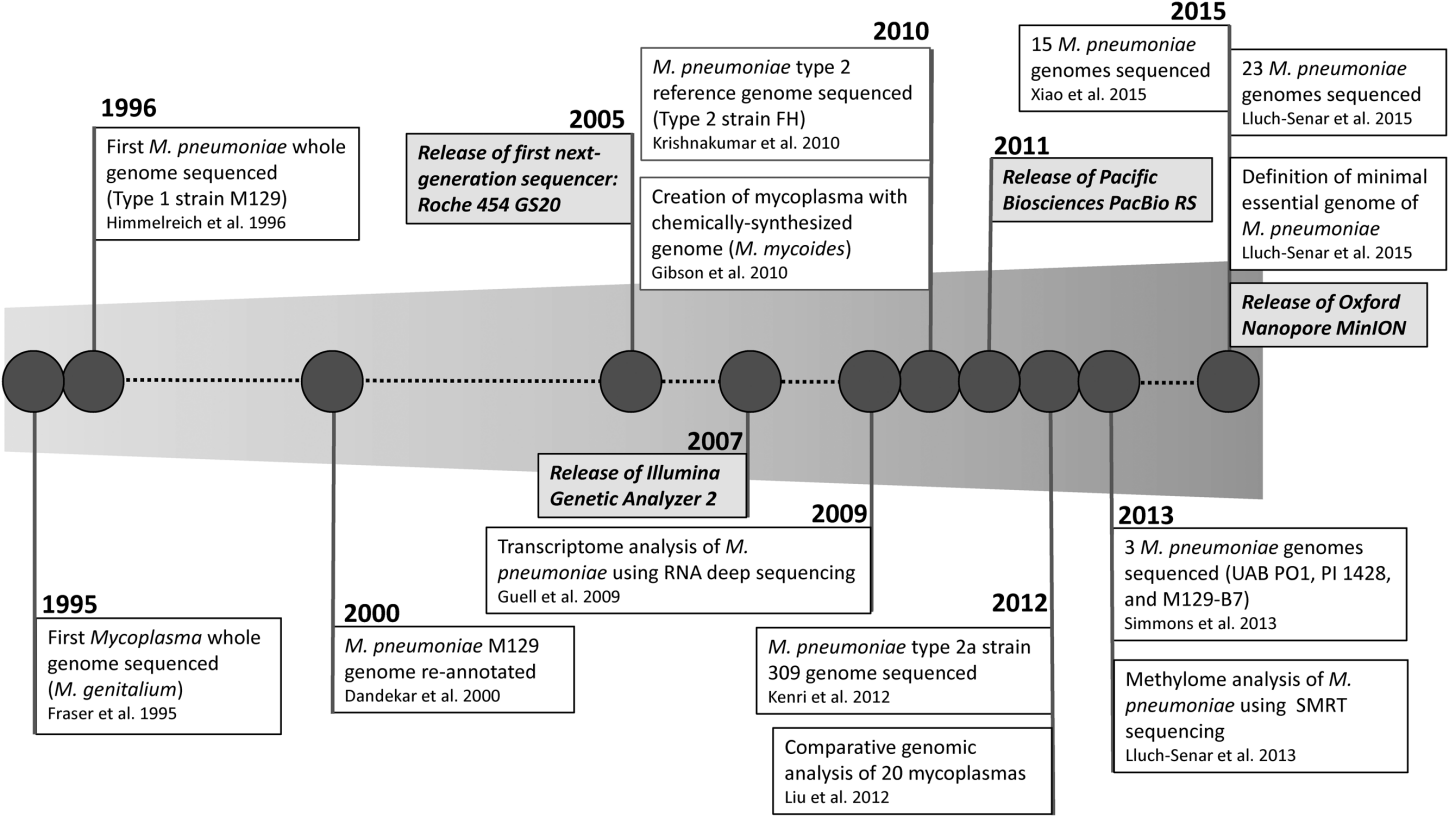
**Timeline of milestones in whole genome sequencing of *Mycoplasma pneumoniae***.

**Figure 1** shows a timeline highlighting the major milestones in *M. pneumoniae* WGS. The genome of *M. genitalium* was one of the first bacterial whole genome sequences obtained in 1995 (Fraser et al., 1995). The *M. pneumoniae* type 1 reference strain M129 followed soon after in 1996, making *Mycoplasma* the first bacterial genus to have whole genome sequences from two different species (Himmelreich et al., 1996). The genome of M129 was subsequently re-annotated in 2000 and found to have 816,394 bp and 730 genes (Dandekar et al., 2000). This served as the only available reference genome for *M. pneumoniae* until the first sequence of a type 2 *M. pneumoniae* strain, the reference strain FH, was reported 10 years later (Krishnakumar et al., 2010). This was followed by the report of the whole genome of a type 2a strain (309) in 2012 (Kenri et al., 2012). Demonstrating the rapid advancement in technical improvements and accessibility to WGS technology, two studies were published in 2015 reporting comparative genomic analysis of 15 and 23 *M. pneumoniae* strains, respectively (Lluch-Senar et al., 2015; Xiao et al., 2015). While a discussion of all “-omics” analysis of *M. pneumoniae* is outside the scope of this review, in the following sections we will discuss recent findings of genomic analyses and how this technology may impact *M. pneumoniae* diagnostics in the future.

### Comparative Genomics

Xiao et al. (2015) analyzed 15 *M. pneumoniae* genomes obtained by Illumina sequencing, including 11 clinical isolates and 4 reference strains. They observed over 99% sequence similarity between all genomes, with the most variation occurring in specific regions within each of the P1 and ORF6 genes, two genes encoded in the same operon which encode components of the adhesin complex. Phylogenetic trees built on whole genome SNP analysis revealed two major clusters based on P1 type. This analysis also suggested that the genome of *M. pneumoniae* is extremely stable over time and geographic distribution, and no evidence of horizontal gene transfer was found in the sequenced isolates (Xiao et al., 2015).

Lluch-Senar et al. (2015) reported a multi-“omics” analysis of *M. pneumoniae*, including comparative genomic analysis of 23 *M. pneumoniae* isolates. Classification of diverse *M. pneumoniae* isolates based on SNPs and indels revealed new subclasses within the broader P1 types 1 and 2 classifications, including four subtypes within type 1 (1a–1d) and five within type 2 (2a–e). The authors concluded that some of these subtypes were associated with country of isolation, but a more comprehensive study including a higher number of isolates representing additional geographic origins is necessary to confirm this observation. Interestingly, this analysis revealed that the frequency of genomic rearrangements was higher than that of SNPs or indels in *M. pneumoniae*. In addition, it was observed that SNPs, indels, and non-synonymous mutations were enriched within genes encoding for proteins involved in virulence, including adhesion molecules. These findings support the purported rearrangement of adhesion genes present in multiple copies within the *M. pneumoniae* genome during infection as a mechanism to circumvent host immune responses (Citti et al., 2010).

Beyond the vast clinical, epidemiological, and microbiological interest in *M. pneumoniae*, it is also commonly used as a model organism in systems biology. The genome of *M. pneumoniae* has been compared to other Mycoplasmas, and the core genome defined for this genus represents the minimal genetic requirements for a prokaryotic organism (Liu et al., 2012). Researchers have even been able to create a viable mycoplasma cell (*M. mycoides*) containing a completely synthetic genome (Gibson et al., 2010). Others have characterized the transcriptome (Guell et al., 2009; Lluch-Senar et al., 2015), proteome (Ueberle et al., 2002; Kuhner et al., 2009; Catrien and Herrmann, 2011; Lluch-Senar et al., 2015), phosphoproteome (Su et al., 2007; Schmidl et al., 2010), methylome (Lluch-Senar et al., 2013), and metabolome (Maier et al., 2013) of *M. pneumoniae*, all of which add to the vastly increasing field of systems biology. The incredibly rapid accumulation of “-omics” data prompted the creation of MyMpn, an open access database for *M. pneumoniae* datasets, including complete genome sequences (Wodke et al., 2015). It is expected that large-scale datasets, including WGS data from many isolates, will continue to grow and be mined for data to investigate *M. pneumoniae* as a pathogen as well as a model organism.

### Impact of Whole Genome Sequencing on *M. pneumoniae* Diagnostics

Whole genome sequencing has the potential to permanently change the field of *M. pneumoniae* biology and epidemiology by allowing improved characterization of strains and better discriminatory power compared to any previous typing method. These data can be used to inform development of newer methods to improve strain discrimination that are accessible to all laboratories. In 2015 alone, two new methods, MLST and SNaPshot mini-sequencing assays, were reported in which whole genome sequence data was used to inform the assay design (Brown et al., 2015b; Touati et al., 2015). Eventually, WGS directly from clinical specimens may become the standard method for determination of etiology of respiratory infections. While the cost of sequencing a bacterial genome has dropped dramatically in recent years, sequencing is still primarily performed on bacterial isolates. Recently, WGS directly from clinical specimens has been demonstrated for detection of respiratory viruses (Zoll et al., 2015) and for *Mycobacterium tuberculosis* (Brown et al., 2015a). Continued technical improvements could allow for direct metagenomics analysis of the entire composition of microbial flora within a patient specimen, which will be critical for implementation of deep sequencing as a primary diagnostic method. This will allow for detailed epidemiological tracking of temporal and geographical trends in strain circulation and will fundamentally change how outbreaks of respiratory disease are investigated.

## Conclusion

Over the past decade, advanced molecular methods for the detection and characterization of *M. pneumoniae* have grown exponentially in regards to both the number and variety of available methods. More widespread implementation of these methods globally has revealed new trends, such as the rapid emergence of macrolide resistance in some parts of the world and the co-circulation of multiple strain types during a discrete period, which challenges a long-standing belief about *M. pneumoniae* epidemiology. Numerous studies in which novel methods were utilized have also highlighted the inadequacy of existing typing strategies, particularly with regards to the inability to definitively link any particular type with clinical characteristics or patient outcomes. The improved accessibility of WGS at the clinical laboratory level and rapidly growing wealth of bioinformatics tools for sequence analysis from clinical specimens is likely to result in a paradigm shift toward WGS analysis for *M. pneumoniae* diagnostics and in clinical microbiology overall.

## Author Contributions

MD and JW contributed to the literature review and interpretation, drafted the work and revised for intellectual content, provided final approval of the version to be published, and agree to be accountable for all aspects of the work.

## Conflict of Interest Statement

The authors declare that the research was conducted in the absence of any commercial or financial relationships that could be construed as a potential conflict of interest. The findings and conclusions in this report are those of the authors and do not necessarily represent the official position of the Centers for Disease Control and Prevention. Use of trade names and commercial sources is for identification only and does not imply endorsement by the Centers for Disease Control and Prevention, the Public Health Service, or the U.S. Department of Health and Human Services.
